# From Cancer to Immune-Mediated Diseases and Tolerance Induction: Lessons Learned From Immune Oncology and Classical Anti-cancer Treatment

**DOI:** 10.3389/fimmu.2020.01423

**Published:** 2020-07-08

**Authors:** Stephan Klöß, Susann Dehmel, Armin Braun, Michael J. Parnham, Ulrike Köhl, Susanne Schiffmann

**Affiliations:** ^1^Fraunhofer Institute for Cell Therapy and Immunology (IZI), Leipzig, Germany; ^2^Institute of Cellular Therapeutics, Hannover Medical School (MHH), Hanover, Germany; ^3^Fraunhofer Institute for Toxicology and Experimental Medicine (ITEM), Hanover, Germany; ^4^Fraunhofer Institute for Molecular Biology and Applied Ecology (IME), Frankfurt, Germany; ^5^Fraunhofer Cluster of Excellence for Immune-Mediated Diseases (CIMD), Frankfurt, Germany; ^6^Institute of Clinical Immunology, University of Leipzig, Leipzig, Germany; ^7^Institute of Clinical Pharmacology, University Hospital Frankfurt, Frankfurt, Germany; ^8^Fraunhofer Institute for Molecular Biology and Applied Ecology (IME), Branch for Translational Medicine and Pharmacology (TMP), Frankfurt, Germany

**Keywords:** immunotherapy, immune tolerance, checkpoint inhibitors, chimeric antigen receptors (CARs), autoimmune disease

## Abstract

Success in cancer treatment over the last four decades has ranged from improvements in classical drug therapy to immune oncology. Anti-cancer drugs have also often proven beneficial for the treatment of inflammatory and autoimmune diseases. In this review, we report on challenging examples that bridge between treatment of cancer and immune-mediated diseases, addressing mechanisms and experimental models as well as clinical investigations. Patient-derived tumor xenograft (PDX) (humanized) mouse models represent useful tools for preclinical evaluation of new therapies and biomarker identification. However, new developments using human *ex vivo* approaches modeling cancer, for example in microfluidic human organs-on-chips, promise to identify key molecular, cellular and immunological features of human cancer progression in a fully human setting. Classical drugs which bridge the gap, for instance, include cytotoxic drugs, proteasome inhibitors, PI3K/mTOR inhibitors and metabolic inhibitors. Biologicals developed for cancer therapy have also shown efficacy in the treatment of autoimmune diseases. In immune oncology, redirected chimeric antigen receptor (CAR) T cells have achieved spectacular remissions in refractory B cell leukemia and lymphoma and are currently under development for tolerance induction using cell-based therapies such as CAR Tregs or NK cells. Finally, a brief outline will be given of the lessons learned from bridging cancer and autoimmune diseases as well as tolerance induction.

## Introduction

In view of the high complexity of the immune system, it is hardly surprising that therapeutic intervention for a disease involving immune dysfunction may result in changes in immune responses that prove beneficial for other immune-mediated diseases. Over the last four decades, this has been the case with therapeutic agents developed for use in cancer—both the early non-selective agents and the recent highly specific biologicals—which are increasingly being found to exert benefit in autoimmune and auto-inflammatory diseases. The resulting commonalities have led to development of new models and approaches to biological therapies covering the whole spectrum of immune responses.

## Classical Anti-Cancer Drugs

### From Anticancer to Autoimmune Disease Therapy

Cytotoxic immunosuppressive drugs go back to the 1950s when cyclophosphamide, an alkylating agent reacting with purine bases to form double-strand adducts which cross-link DNA to trigger apoptosis, was introduced for the therapy of solid and hematological malignancies. Because of its immunosuppressive activities, cyclophosphamide has subsequently been used for the treatment of systemic lupus erythematosus, vasculitis and other autoimmune diseases, but its non-specific cytotoxicity severely restricts its clinical use, a common limitation for the broader use of many anti-cancer agents (1).

Around the same time, several antimetabolites were developed for use in cancers, the agent subsequently used to the greatest extent being methotrexate (Table 1). This drug is a folate analog which inhibits the enzymes dihydrofolate reductase and thymidylate synthase, thereby depleting tumor cells of the purine and pyrimidine precursors required for DNA and RNA synthesis (23). The subsequent history of methotrexate use, well illustrates the courses of a number of drugs introduced for cancer therapy which have found applications in other diseases. It was initially found to be of use in psoriatic arthritis and in this autoimmune disorder continued to exert therapeutic efficacy at doses considerably lower than those required in cancer. Michael Weinblatt overcame the widespread reservation about using an anti-cancer drug for autoimmunity and performed randomized controlled trials with methotrexate in rheumatoid arthritis (RA) (24). The drug has long since become the standard of treatment for rheumatoid arthritis, but at the relatively low doses used, its mechanism of action is thought to be due to enhanced conversion of AMP to extracellular adenosine, an endogenous anti-inflammatory substance which reduces macrophage cytokine release. Recently, though, it has been shown to inhibit JAK1/2 kinases, which are involved in inflammatory cell signaling (25).

**Table 1:** Overview of drugs used for oncological and immunological indications.

**Target**	**Drugs**	**Oncological use**		**Immunological use**	
		**Indications**	**Potential mechanism**	**Indications**	**Potential mechanism**
Dihydrofolate reductase/thymidylate synthase	Methotrexate	Breast cancer, leukemia, lung cancer, lymphoma, osteosarcoma (2)	Antimetabolite, depletes tumors of precursors for RNA/DNA synthesis (2)	Psoriasis, rheumatoid arthritis (3)	Conversion of AMP to extracellular adenosine; JAK1/2 kinase inhibition (3)
CD20	Rituximab, Ocrelizumab	B-cell non-Hodgkin's lymphoma, B-cell chronic lymphocytic leukemia (Rituximab) (4)	B cell depletion by induction of apoptosis, antibody dependent cellular cytotoxicity (ADCC), complement dependent cytotoxicity (CDC) (5)	Multiple sclerosis (Ocrelizumab) (6), severe refractory systemic lupus (Rituximab) (7), ANCA-associated vasculitis (Rituximab) (7), RA (Rituximab) (7)	B cell depletion by induction of apoptosis, antibody dependent cellular cytotoxicity (ADCC), complement dependent cytotoxicity (CDC) (8)
Proteasome	Bortezomib	Multiple myeloma (9)	Induction of apoptosis and inhibition of tumor cells, reduction of cytokine and VEGF production (10)	Potential use for myasthenia gravis, severe SLE (11, 12)	Induction of apoptosis of plasma cells, reduction of cytokine production (13)
PI3K/mTOR	Everolimus, Sirolimus, Temsirolimus	Advanced renal cell carcinoma (14), gastroeneropancreatic neuroendocrine tumor (15), subependymal giant cell astrocytoma (16), breast cancer (17)	Reduction of cell growth and proliferation by inhibition of mTOR pathway (18)	Renal transplantation to prevent organ rejection (19)	Suppression of T cell proliferation by inhibition of mTOR pathway (20)
IDH	Enasidenib	Acute myeloid leukemia (21)	Inhibition of 2HG synthesis (22)	Not identified yet	

### Rituximab

Rituximab (Table 1) was one of the first therapeutic monoclonal antibodies to be introduced to the clinic in the 1990s. Directed toward CD20 on the surface of B cells, its selective efficacy at four weekly doses of 375 mg/m^2^ in non-Hodgkin lymphoma (NHL) of B cell origin is based on the fact that CD20 is expressed on both healthy and NHL B-cells, but not on immature or developing B cells (26). With a long half-life, rituximab can be found in plasma and bound to circulating B cells for up to 6 months, making it useful for treatment of chronic diseases (27). Thus, from the outset of its development, despite the fact that significant decreases in circulating immunoglobulins were not observed in lymphoma studies, there was considerable interest in studying rituximab for B cell depletion in autoimmune diseases in which generation of autoantibodies is a major pathological issue. Initial studies were carried out in IgM-associated polyneuropathies associated with a lymphoblastic B cell clone in the bone marrow which has a low proliferation rate and is not susceptible to conventional immunosuppressive but expresses CD20 (28). Intriguingly, in multi-morbid patients with lymphoproliferative diseases, beneficial effects of rituximab were also observed on autoantibody-related autoimmune manifestations. Following the discovery by Edwards and Cambridge in 1998 that auto-reactive B cell clones are promoted by macrophage activation and inflammation, clinical trials were initiated in RA (29). Rituximab in combination with methotrexate was licensed for use in RA in 2006 at 2 × 1,000 mg separated by 2 weeks. It has subsequently been licensed for ANCA-associated vasculitis and severe refractory systemic lupus (SLE). B cells and the generation of autoantibodies are also major players in the development of multiple sclerosis (30, 31). Consequently, rituximab also showed efficacy in the treatment of multiple sclerosis, leading to the development of ocrelizumab (Table 1) a humanized antibody directed toward CD20 that was approved for the treatment of multiple sclerosis patients (6, 32).

The realization that rituximab has clear efficacy in various inflammatory and autoimmune diseases, sparked off a search for other drugs which could selectively modulate B cell function. Notable among these is belimumab, which binds to B cell activating factor (BAFF) or B-lymphocyte stimulator (BLyS). This mediator is required for the normal development and survival of B cells. In SLE and also multiple sclerosis patients, however, BAFF is overexpressed, contributing to autoimmune B cell proliferation (33). Binding of belimumab to BAFF prevents it from binding to autoimmune B cells, resulting in B cell apoptosis (34). Belimumab was introduced for the therapy of SLE in 2011, the first new drug specifically approved for this indication in 56 years. A variety of follow-up drugs are under development (35).

### Bortezomib and Proteasome Inhibitors

Bortezomib (Table 1), a dipeptide boronate, is a selective inhibitor of the 20S proteasome, a subunit of the 26S proteasome, which degrades intracellular proteins labeled by linear ubiquitination for subsequent hydrolysis of the peptides generated (36). Its development arose out of research led by Alfred L. Goldberg into the role of protein breakdown in the muscle wasting or cachexia seen in many systemic diseases such as cancer, sepsis and renal failure. The discovery of the role of the proteasome in the activation of the key transcription factor, NFκB, diverted the research toward development of anti-inflammatory, anti-neoplastic compounds (36). Inhibition of NFκB prevents apoptosis in tumor cells with a high protein turnover, causes ER stress and as a result of proteasome inhibition, misfolded proteins accumulate intracellularly (37). Based on these effects, bortezomib was approved for the treatment of multiple myeloma in 2003. Several other proteasome inhibitors are also under development for oncological indications (38).

Inhibition of intracellular protein degradation also modifies antigen presentation and the generation of antibodies, including autoantibodies through inhibition of the immunoproteasome, a specialized form of proteasome, mainly expressed in lymphocytes and monocytes. Consequently, antibody-producing plasma cells, which also have high protein turnover, are sensitive to inhibition by bortezomib and experimental studies suggest its potential use in the treatment of the autoimmune diseases, myasthenia gravis (MG) and severe SLE (11). A number of cases have been reported in which bortezomib was tested clinically. Currently a prospective, non-randomized clinical trial is in progress in which bortezomib is being tested in MG, SLE and RA patients refractory to current therapeutic regimes (12). Unfortunately, cells adapt to survival in the presence of proteasome inhibitors and other approaches are being taken to inhibit different types of proteasome complexes found within cells (38). One such approach involves inhibitors of the E3 ligases involved in ubiquitin activation and one, pevonedistat (MLN4924) has already entered clinical trials for acute myeloid leukemia (39). Many research groups are developing PROTACs (Proteolysis Targeting Chimeric Molecules), bispecific molecules which both act as ligands for E3 ligase and bind to the target protein to be tagged with linear ubiquitin for degradation by the proteasome (40). This would be of benefit both for tumor-targeted therapy and potentially for the inhibition of autoantibody production.

### PI3K/mTOR Inhibitors

Mammalian target of rapamycin (mTOR), the downstream effector of phosphatidylinositol-3-phosphate kinase (PI3K), is a component of the epidermal growth factor receptor (EGFR) signaling pathway induced by natural ligands such as EGF, leading to cell growth and proliferation. The mTOR-AKT-PI3K pathway is dysregulated in many cancers (41). Everolimus, sirolimus (rapamycin) and temsirolimus (Table 1) inhibit mTOR and thereby cell proliferation. In this context, everolimus and temsirolimus showed efficacy in the treatment of advanced renal cell carcinoma (RCC) (14). Everolimus is also approved for the treatment of gastroenteropancreatic neuroendocrine tumor (15), subependymal giant cell astrocytoma (16) and breast cancer (17). Everolimus and sirolimus are further approved for prevention of organ rejection after renal transplantation, since inhibition of the mTOR pathway suppresses T cell proliferation. However, mTOR inhibition also increases the capacity of phagocytic cells to release cytokines such as IL-12 leading to the priming of pro-inflammatory TH1 and TH17 responses (20). Thus, the inflammatory side effects that can occur in transplant recipients treated with rapamycin are possibly due to this interaction with cytokine release by phagocytic cells. Another severe adverse outcome of transplantation is malignancy, a major cause of post-transplant mortality. Since mTOR inhibitors exert various anti-proliferative effects, transplant patients suffering from such malignancies can benefit from both the immunosuppressive and the anti-carcinogenic potential of mTOR inhibitors. In keeping with this, a lower rate of *de novo* malignancy under mTOR inhibition after solid organ transplantation has been observed (42, 43). Everolimus is also effective in therapy-resistant autoimmune hepatitis (44) and given in combination with methotrexate, it provides clinical benefit in RA (45), but is not approved for these indications.

### Metabolic Inhibitors

The incentive to develop effective, more potent and less toxic drugs stimulated the search to identify pathways that are critical for the survival of, or even exclusive use by cancer cells. In this respect, isocitrate dehydrogenase (IDH) enzymes were identified since they normally metabolize isocitrate to α-ketoglutarate. In a mutated state—as found in AML patients and in low-grade gliomas—IDH also converts α-ketoglutarate into the oncometabolite 2-hydroxyglutarate (2HG) that causes cell differentiation defects by impairing histone demethylation (22). Enasidenib (Table 1), a first-in-class inhibitor of mutated IDH2, was approved for the treatment of acute myeloid leukemia (AML) (21). In addition, immunometabolism-modulating drugs that can improve immune cell survival or modify the interactions between cancer cells and immune cells have become a focus of investigation. Epacadostat, an indoleamine 2, 3-dioxygenase 1 (IDO1) inhibitor, controls tryptophan metabolism to foster immune cell activity. However, epacadostat in combination with pembrolizumab failed to provide superior outcome in melanoma when compared to pembrolizumab alone (46). In contrast to the other drugs discussed in this review, the use of these metabolism-modifying anti-tumor agents for autoimmune diseases is in its infancy. It is questionable whether IDH inhibitors are suitable for the treatment of autoimmune diseases since metabolic inhibition could lead to a decrease in immune cell activity, although metabolic interactions can significantly modify the inflammatory status of immune cells. Pro-inflammatory immune cells such as macrophages, for instance, are characterized by upregulated glycolysis, impairment of oxidative phosphorylation, and disruption of the Krebs cycle at two steps, after citrate and succinate formation (47). Citrate is used in fatty acid biosynthesis, which permits the increased synthesis of inflammatory prostaglandins. Succinate activates the transcription factor HIF-1α, a regulator of a wide range of genes, including IL-1β, CCL2, and CXCL8 (48–50). The inhibition of IDH could lead to an increase in citrate, potentially accompanied by an increase in inflammatory prostaglandins and to a decrease in succinate. This is potentially linked to a reduced synthesis of pro-inflammatory cytokines and to inhibition of glycolysis, possibly accompanied by a shift in immune cells toward a more anti-inflammatory status. However, further studies are needed to investigate whether metabolic inhibitors are suitable for the treatment of autoimmune diseases.

### Lessons Learned

**Figure 1:**
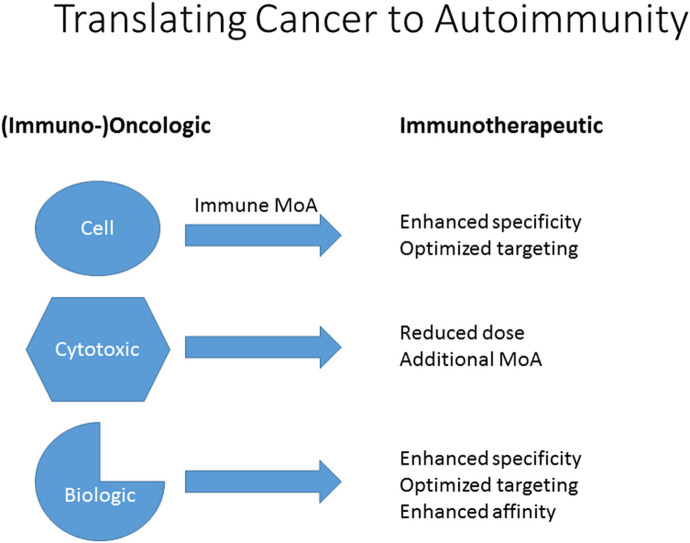
Translation of cellular, cytotoxic and biologic agents from (immuno-) oncological to immunotherapeutic use in autoimmunity. Clarification or discovery of mechanisms of action (MoA) will assist in optimizing dosing regimens, improve specificity and targeting and facilitate repurposing.

The development of cytostatic anti-tumor agents for use in autoimmune diseases such as psoriasis and RA emphasizes the importance of careful dissection of the (broader) mechanisms of action of drugs which modulate immune responses, particularly those mechanisms that are not immediately relevant to the targeted oncological indication. These include intracellular signaling processes, but also cell growth, metabolic and cell surface binding interactions. This is not only crucial for an understanding of the breadth of pharmacological activity of these agents, but for their potential repurposing for other important immune disorders and also for potential immunotoxicity. Thus, to translate cytotoxic, biological and cellular agents from oncology to autoimmune applications, clarification of their mechanisms can lead to dosing improvements, novel targets and unexpected uses (Figure 1). In the following, some examples are provided.

Rituximab is a prime example of increased understanding of both the mechanism of action on B-cells and their role in different autoimmune diseases opening up totally new markets for the drug and for a whole new class of B cell inhibiting drugs, including belimumab. This class is likely to be extended with proteasome inhibitors, such as bortezomib, which are effective in myeloplasias and appear to bear promise for treatment of diseases in which autoantibody generation is high. Undoubtedly, with the widespread efforts to identify novel immune-oncological drugs and new targets for modulation of immune-mediated diseases, there will be an increased dove-tailing of research programs to identify targets, such as the well-characterized PI3K/mTOR inhibitors, which find parallel therapeutic applications for both cancer and inflammatory and autoimmune disorders.

The broad ramifications for immune-mediated disease therapy of drugs developed as immunotherapies for cancer are well illustrated by immune checkpoint inhibitors, such as those acting at PD-1. These have been extensively discussed in a recent review (51). Shown to be active in a variety of cancers, including melanoma, metastatic lung cancer, kidney cancer and Hodgkin's lymphoma, agents targeting PD-1 or PD-L1 have also been found effective for lupus, psoriasis and inflammatory bowel disease, as well as being investigated for potential use in chronic infection and sepsis (51, 52). PD-1-related immune checkpoint inhibitors also illustrate the complications that arise with the pharmacological modification of immune homeostasis, such as skin, renal and hepatic toxicities.

The development of anti-cancer drugs for immune-mediated diseases thus, highlights the relevance of altering the dosing regimen to reduce potential anti-tumor-related toxicity, but retaining therapeutic effects in inflammatory or autoimmune conditions. In translating immunotherapeutic agents from cancer therapy to treatment of chronic inflammation and autoimmunity, toxicities are inevitably less acceptable. Understanding mechanism of action (MoA) of methotrexate at lower doses led to substantial reduction in toxicity while applying this drug.

mTOR is a good example of a target which has experienced “indication-hopping,” having been developed for immunosuppression and immunomodulation and then as an anti-cancer and inhibitor of cellular senescence. A recent report indicates that doses of everolimus can also be readjusted, depending on the indication (cancer or transplant rejection), to reduce unnecessary toxicity (53). The further demonstration that everolimus, like rapamycin, can slow immunosenescence in the elderly suggests that a downward readjustment of the dose may result in a well-tolerated dosing regimen in chronic immune-mediated disorders in the elderly (54).

Another illustration of an agent developed at a high dose for cancer treatment which was subsequently pursued at a low dose for immune-mediated diseases is the cytokine interleukin-2 (IL-2). Recombinant IL-2 was first developed as a stimulant of T cell immunity by administration at a high dose with autologous lymphokine activated killer cells for the treatment of metastatic melanoma and kidney cancer. Subsequently, it was found that Treg cells express the IL-2 receptor CD25 constitutively, and that IL-2 is more critical for the development and survival of Tregs than for effector T cell function (55). This discovery has given a pronounced incentive to the development of drugs acting at CD25 on Tregs for the treatment of immune-mediated diseases. In the future, we should expect to see drug companies seeking parallel development of immunotherapies for various indications instead of the classical development for a primary followed by a secondary indication. “There is clearly a strong rationale for further expanding the opportunities for cross-fertilization of ideas and approaches between cancer immunology and autoimmunity, so that further synergies between the two fields can accelerate the development of effective immunotherapies”(55).

## Cell-Based Therapies

### From CAR T Cells in Immune Oncology to CAR Tregs for Tolerance Induction in Immune Mediated Disease

#### CAR T Cells

Chimeric antigen receptor (CAR) modified T cells are a novel class of anti-cancer therapy for target-specific recognition and destruction of cancer cells. An extracellular single-chain variable fragment (scFv) antibody is used to bind to the respective cancer target combined with an intracellular CD3zeta chain to activate T cells (56). Linking to a second co-stimulatory domain results in lasting T cell response and prolonged cell survival. The first five generations of CARs have been reviewed (57, 58).

Adaptive immunotherapies using these CAR T cells have achieved spectacular remissions in refractory B cell leukemia and lymphoma. So far, frequent, durable and objective regression in pediatric B cell acute lymphoblastic leukemia (B-ALL), chronic lymphocytic leukemia (CLL), and B cell lymphoma have been reported using anti-CD19 CARs (59–62). In 2017 and 2018, two CD19 CAR T cell products (Tisagenlecleucel (Kymriah®), Axicabtagene ciloleucel (Yescarta®) received marketing approval in USA and Europe, respectively. Treatment cost is >275.000€/product and patient. Clinical trials with CAR T cells in several malignant diseases now constitute a fast-growing field with >1,000 clinical trials registered with clinicaltrials.gov, most of them undertaken in the United States and China and around 10% in Europe. While most of the clinical trials still address hematological malignancies, the number of trials in oncology is increasing continuously (63–65). Management of the severe side effects, such as cytokine release syndrome or neurotoxicity, which appear in 2/3–3/4 of the patients, has been established and reviewed (66).

For the increasing numbers of patients, the reproducible manufacture of high-quality clinical-grade CAR T cell products is becoming a growing challenge, moving from manual to a more automated process (67–70). In Europe, CAR T cell manufacturing is regulated by the Tissue and Cell Directives published in 2004 (2004/23/EC) and 2006 (2006/17/EC; 2006/86/EC), respectively. Beside autologous CAR T cells for individualized medicine, initial studies have been performed using allogeneic “off the shelf” CAR T cells from healthy donors (71).

#### CAR Tregs

Tolerance induction is a major goal in cell-based immunotherapy. Gene-modification of CAR Tregs has provided significant advantages with clinical applications in organ transplantation and cell therapies. Early clinical studies recently demonstrated the tolerability, safety, broad spectrum of effects and feasibility of Treg-based cell therapies for excessive immune reactions, such as GvHD, or autoimmune diseases, tissue protection and to prevent progression of inflammatory disorders (72). In particular, new technologies for the production of CAR Tregs with selective potential against aggressive effector cells, reflected by an excessive T cell response and autoimmune reaction, can be attenuated by specific CAR Treg cell activity (72–74). Initially, CARs were used in 2 subgroups of CD28-CD3ζ CAR-modified Tregs, which were redirected against the carcinoembryonic antigen (CEA). This surface target is often overexpressed on human lungs as well as in the intestine, in colon cancer and colonic inflammation (ulcerative colitis) (75–77). Other studies revealed that human CD19-engineered CAR Tregs were able to suppress the cytotoxicity and proliferation of CD19 CAR T effector cells *in vitro*. Mouse tumor (CD19+) experiments demonstrated clearly that tumor-infiltrated CD19-modified CAR Tregs inhibited CD19 CAR T cell-dependent tumor elimination at a ratio of 1 (CAR-Tregs) to 16 (anti-CD19 CAR T effectors) (78). Recently, systematic testing of humanized HLA-A2 CARs revealed their ability to interact with HLA-A^*^02:01 and to trigger human Treg-mediated suppression *in vitro*. Moreover, transplantation of human HLA-A2-CAR Tregs inhibited HLA-A2-positive effector cell-associated xenogeneic GvHD and decreased rejection of human HLA-A2-positive skin allografts (72, 79). These results suggest the use of humanized alloantigen-specific CARs to engineer retargeting and specificity of clinically applicable Tregs.

### Role of NK Cells in Cancer and Autoimmune Disease

Human natural killer (NK) cells (~10% of PB lymphocytes) are an important subpopulation of innate lymphoid cells (ILCs), which play an essential role in innate defense against virally infected and cancer cells (80–82). Their activation is controlled by a highly sensitive balance between natural cytotoxicity receptors (NCRs) and killer cell immunoglobulin-like receptors (KIRs) responsible for recognition of “non-self” transformed cells without major histocompatibility complex (MHC) or specific antibodies (80, 83, 84). Broad cytotoxic mechanisms and rapid stimulation of immune reactions make this lymphoid cell type suitable as a candidate for use in cancer immunotherapy. In the last decade, a strong focus has been laid on the establishment and validation of chimeric antigen receptor (CAR)-modified effector cells to treat refractory cancer patients but mainly using autologous T cells as a source of potent effector cells. Unlike T cells, NK cells lack the potential to generate graft-vs.-host disease (GvHD) and the absence of this adverse response makes NK cells an ideal alternative to CAR-modified T cells (81). This potentially improved safety of engineered CAR NK cells for cancer immunotherapies, in comparison to CAR T cell therapies, could stimulate broad research and development in the field of cancer immunity (81, 85). CAR-modified NK cells thus represent a potential source of combined gene- and cell therapies, offering potential allogeneic “off-the-shelf” cellular therapy mediating severe anti-leukemic and anti-tumor effects without triggering potentially lethal alloreactivity such as GVHD.

In addition to their ability to fight cancer cells in a targeted and effective manner, NK cells also seem to have immunomodulating, protective properties. Accordingly, allogeneic NK were advantageous in patients with mismatched hematopoietic transplants by dint of their strong graft-vs.-leukemia (GvL) effects and amelioration of leukemia relapses, but also by protection of these patients against GvHD and graft rejection (80, 86, 87). NK cell-dependent immunotherapies largely prevented transplant rejection by sustaining the hematopoietic transplant and exerting a GvL response (80, 88).

The important function of NK cells in autoimmune disease remains to be fully clarified (83, 89). Past studies have provided multiple indications that certain subgroups of NK cells probably exercise a protective mechanism to counteract autoimmune diseases. In this context, distinct NK cell subsets were repeatedly reported to result in a clear attenuation of the overall Th1 response in autoimmune diseases by releasing Th2 cytokines (89). Moreover, NK cells are able to down-regulate the CD4 and CD8 T cell response during chronic viral infections by binding, in particular, of TNF-related apoptosis-inducing ligand (TRAIL) or by secretion of high perforin levels to induce T cell apoptosis (90–92).

A protective effect of NK cells could also be demonstrated in patients with multiple sclerosis (MS) (93, 94), high surface expression levels of CD95 (Fas) being detected on NK cells derived from patients in disease remission which were classified as “NK2” cells. These NK cells secreted high amounts of interleukin-5 (IL-5) and IL-13 (94, 95). Interestingly, NK cells isolated from patients with MS exhibited lower proliferation capacity and restricted effector cell functions (96). One hypothesis suggested that activated NK cell subsets are mainly responsible for decreased production rates of interferon-gamma (IFNg) in resident effector/memory T cells. Accordingly, *ex-vivo* experiments with NK cell-depleted PBMNC showed enhanced IFNg levels after stimulation of T cells which underlines the regulatory role of NK cells in MS (94).

In experimental murine autoimmune encephalomyelitis (EAE), CNS inflammation was abolished and spinal cord and brain damage attenuated by transferring acetylcholine-producing NK cells into the cerebral ventricles which suppressed infiltrating/resistant macrophages and monocytes (97, 98). In contrast, increased inflammation levels were detected after depletion *in vivo* of these NK cells. Experiments *in vitro* showed increased CD4 T cell frequencies followed by enhanced Th1 cytokine secretion as a result of NK cell depletion (98).

Recent studies have shown that the adoptive transfer of CXCR5-negative NK cell subsets improves autoimmune myasthenia gravis (EAMG) symptoms by down-regulation of splenic follicular helper T (Tfh) cells and germinal center B cells, inducing apoptosis of T cells but not of B cells. CXCR5-negative NK cells were found mainly outside the B cell zone, whereas CXCR5-positive NK were localized within the B cell zone and secreted higher IL-17 levels. These data suggest that a distinct (CXCR5-negative) NK cell subset is responsible for inhibition of the autoimmune response in EAMG models (99).

Despite these encouraging results from scientific studies, no data are available from controlled prospective studies. There is still no clear explanation of the role of NK cells in autoimmunity, and further studies are necessary to characterize distinct NK subsets, how they exacerbate inflammatory reactions and which key NK players protect against the progression of excessive inflammation.

### Mesenchymal Stem Cells in the Treatment of Autoimmune Diseases

Mesenchymal stem cells (MSC) are a heterogeneous group of multipotent, non-hematopoietic, self-renewable progenitor cells of different cell types which can differentiate into adipocytes, chondrocytes, osteoblasts and myocytes (100–102). Because this type of stem cell has potent immunosuppressive effects on both the innate and acquired immune system, MSCs have been used therapeutically in the last two decades for their immunomodulatory effects and their seemingly low toxicity and side effects in various autoimmune diseases. During this period, thousands of patients were treated with autologous and even allogeneic MSCs for the targeted treatment of various diseases and a large number of clinical studies (see clinicaltrials.gov) have tested the effectiveness and feasibility of MSC-based therapies under clinical conditions, including GvHD, Crohn's disease, rheumatoid arthritis (RA), systemic lupus erythematosus (SLE), MS, Type 1 diabetes mellitus (T1DM), organ (kidney) transplantation, cardiovascular diseases, neurological diseases, hematological malignancies and autoimmune diseases (101, 103–105).

Despite advances in the research and development of novel treatments and biological agents, successful treatment of autoimmune diseases remains unattainable. Recently, both the therapeutic benefit of MSCs and their capacity to counteract autoimmune disease progression was reported (106, 107). The immune-modulating effects of MSCs on other lymphoid and myeloid cell types is mediated by the multiple release of mediators, including transforming growth factor beta (TGF-β), prostaglandin E_2_ (PGE_2_), nitric oxide (NO), soluble HLA-G or indoleamine 2, 3-dioxygenase (IDO). Such effects also occur in the presence of increased plasma levels of tumor necrosis factor alpha (TNFa), toll-like receptor 3 (TLR3) agonists and IFNg (107, 108). As a result CD4+CD25+CD127– and CD4+CD25+Foxp3+ regulatory T cell (Tregs) subsets are stimulated, resulting in enhanced immunosuppression of cytotoxic CD8+ T cells and CD56^dim^CD16+ NK cells (107–110).

A well-studied example of efficacy in the treatment of autoimmune diseases in patients is in systemic lupus erythematosus (SLE), a chronic autoimmune disease with clinical manifestations in all organs of the body, associated with increased morbidity and mortality (111, 112). A clinical study with allogeneic umbilical cord MSCs demonstrated the safety and effectiveness of MSC therapy in refractory SLE patients (113–115). Previous studies in refractory and severe SLE patients revealed a tendency toward clinical remission and an amelioration of serological markers for organ dysfunction (100, 112, 116, 117). Interestingly, only allogeneic MSCs from healthy donors, but not from autologous SLE patients, showed immunosuppressive properties in SLE patients while improving symptoms of SLE disease. Moreover, more precise characterization of patient-derived MSCs indicated phenotypical senescence and a number of dysfunctions in immune regulation and proliferation (113). These data were confirmed in another clinical study in which, after allogeneic MSC transplantation from healthy donors, only the proportion of refractory SLE patients showed clinical remission or extenuated disease symptoms. Other SLE patients did not benefit from an autologous MSC transplantation approach (118), which suggests that MSCs derived from SLE patients have several immunosuppressive dysfunctions. At the present time, nine clinical study protocols can be found for MSC-based treatments of SLE patients (www.clinicaltrials.gov).

MSC-containing transplants have also been successfully performed in the treatment of Crohn's disease, a chronic inflammatory reaction of the gastrointestinal tract. Accordingly, improvement in the disease course was achieved in three of eight MSC-transplanted patients with refractory Crohn's disease following autologous bone marrow-derived MSC transplant. However, five of these eight patients showed an ameliorated Crohn's disease activity index score (100, 119). Complete occlusion of the fistula tract with a simultaneous reduction in the activity index for Crohn's disease and healing of the rectal mucous membranes was observed in the majority of the patients within 1 year (100, 120). This could be confirmed in further long-term observations of the same patients (100, 121). To date, almost twenty clinical studies are available on MSC transplantations in Crohn's disease at different stages listed in the online database (www.clinicaltrials.gov).

Due to the wide clinical application of MSC-based immunotherapies in autoimmune diseases, this innovative research field has also been expanded to investigate the primary immunomodulatory effects of MSCs in more detail. Recent studies demonstrated that release of extracellular vesicles, especially of so-called exosomes, represents an important mechanism of action of MSCs which weakens the symptoms of autoimmune diseases (107, 122). These hypoimmunogenic, blood-brain-barrier-crossing vesicular carriers for intercellular communications contain high amounts of immunoregulatory molecules to trigger self-tolerance. Thus, the MSC-derived extracellular vesicles (MSC-EVs) contain mRNAs, microRNAs (miRNAs), cytokines, chemokines and immunomodulatory factors that seemed to down-regulate chronic inflammation or infections (122). Recently, it was demonstrated that MSC-EV-mediated efficacy was largely equivalent to the immunosuppressive effects seen after the transplantation of MSCs into patients with autoimmune disease. Moreover, MSC-EV-mediated effects were detected in some autoimmune and inflammation mouse models, as in the protection of hepatocytes in acute liver injury and fibrosis, in the treatment of lung inflammatory diseases, in attenuation of neuroinflammatory and inflammatory eye diseases, in the protection of renal tubular epithelial cells and injured cardiomyocytes (122). In summary, MSC-EVs exert immunosuppression and represent a potentially novel therapeutic remedy.

## Preclinical Immune Competent Models for Drug Development of Immunomodulatory Drugs

### Definition of the Problem

Healthcare is evolving from reactive disease care to care that is predictive, preventive, personalized and participatory. Selecting and developing the optimal drug for each patient requires both profound understanding of cancer molecular biology, as well as well-established immune competent pre-clinical tests. Being able to transfer results from the lab to clinical studies and beyond is crucial Mimicking the immune system of a human being that has usually evolved over decades in its interaction with a unique environment, dealing with multiple provocations like infections, pollutions etc., is extremely challenging. In order to mimic a realistic human immune response and subsequently allow for the development of immunomodulatory strategies for treatment of cancer and autoimmune diseases, several strategies have been proposed. These involve humanized mouse models and immune competent, human-based *ex-vivo* models (123). In this section, we provide a broad overview of patient-derived, immunocompetent preclinical models, their applicability in drug development and personalized medicine, as well as their advantages and disadvantages.

### Humanized Mouse Models for Cancer

Patient-derived, tumor xenograft (PDX) (humanized) mouse models represent the classical tools for systemic preclinical evaluation of new therapies and biomarker identification. Within the past decade, cancer chemotherapy has evolved from non-specific drugs that damage both tumor and normal cells, to more specific agents and immunotherapeutic approaches, which have shown greater effectiveness with less toxicity (124). The understanding of the molecular pathogenesis of cancer, particularly understanding of the critical importance of complex interaction of tumor cells with tissue resident cells, has increased remarkably. This has led to a dramatic increase in the number of experimental agents and clinical trials for human cancers. Unfortunately, our preclinical models perform poorly as predictive platforms for the ultimate success of clinical candidates, reflecting the complexity of cancer (125). The new class of immune modulating drugs, like immune checkpoint inhibitors or cellular therapies such as CAR T cells, require the development of predictive, immune competent preclinical models (125, 126).

CAR-engineered T cells have been largely successful in treating hematological malignancies in the clinic. Unfortunately, CAR T cell therapy can cause dangerous side effects, including off-tumor toxicity, cytokine release syndrome, and neurotoxicity. Animal models of CAR T cell therapy often fail to predict such adverse events and frequently overestimate the efficacy of the treatment. Nearly all preclinical CAR T cell studies have been performed in mice, including syngeneic, xenograft, transgenic, and humanized mouse models. Syngeneic or immunocompetent allograft mouse models use CAR T cells, tumors, and target antigens that are all murine derived (127, 128). Many CAR T cell studies are done in human xenograft models, where it is hard to delineate between xenogeneic rejection, allogeneic response of human CAR T cells to the tumor, and the actual CAR T cell therapeutic efficacy in causing tumor regression. Furthermore, the lack of host immune system does not allow testing of the TME, the tumor's metastatic potential, or the host response to CAR T cells. Only a few studies have used xenograft mice to study the effects of Tregs on CAR T cell efficacy, but studies including other immunosuppressive cells are lacking. The syngeneic or immunocompetent allograft mouse models use CAR T cells, tumors, and target antigens that are all murine derived. However, the syngeneic model has its drawbacks, as mouse biology does not always accurately recapitulate human biology. For example, murine immune systems differ from that in humans, and syngeneic models have been largely unable to mimic CRS (128). However, several very successful drug developments have been based on murine cancer models. Humanized mouse models reflecting parts of human immune responses can be used. Patient-derived xenograft (PDX) mouse models (NOD, Prkdc^scid^, and Il2rγ^−^) were developed (129) and used for checkpoint inhibitor studies. For example, BALB/c-Rag2nullIl2rγnullSIRPαNOD (BRGS) pups are humanized through transplantation of cord blood (CB)-derived CD34+ cells in order to test anti-PD-1 immunotherapy (130). Recently, the limitations of these models became clearer. The genetically and/or immunological modified laboratory mouse, transplanted with a cultured tumor cell line or primary isolated tumor cells, has been the predominant preclinical model used to assess potential therapeutic efficacies. However, these mouse models often do not adequately reflect tumor progression and the cellular, immunological and genetic heterogeneity found within human cancers. Furthermore, laboratory mice also present with a vastly restricted immune profile compared to humans (131).

To address the failure rate of clinical trials in oncology, preclinical models that accurately predict clinical outcomes are urgently needed. Therefore, the so-called “Avatar” concept for co-clinical trials has emerged. PDX Avatar *in-vivo* models are generated from the tumors of patients enrolled in a clinical trial, and these models are treated simultaneously with the same agents administered to the patients in the clinical trial. Coupled with tumor genomic profiling data, Avatar co-clinical trials are designed to aid in the design of personalized therapeutic regimens in real time (132).

### Human *ex vivo* Models for Cancer and Autoimmune Disease Models

The Avatar concept is also applicable to *in vitro* and *ex vivo* models (133). This article focusses on *in vitro* and *ex vivo* patient-derived models with increasing tumor heterogeneity and complexity and describes the application of the models in drug research and development.

*In vitro* cancer models extend from commercially available cancer cell lines to patient-derived primary disseminated cancer cells, which can be used to generate patient-derived cancer cell lines (PDCL). The most widely used preclinical models are conventional cell lines, such as the NCI-60 standard panel developed in the late 1980s (134). However, the accumulation of genetic aberrations in cancer cell lines with increasing passage number (135) and the lack of tumor heterogeneity highlight the limitations of cell line-based models and pave the way to patient-derived cell models. Patient-derived tumors are dissociated enzymatically and/or mechanically or circulating tumor cells are isolated from blood as a biological correlate of metastasis (136–139). These slow proliferating, dissociated tumor cells exhibit the heterogeneity of the original tumor and are known to be of prognostic relevance (123). Unfortunately, establishment of cell lines from these primary tumor cells is inefficient and often requires cycles of re-implantations as xenografts in mouse models. Still some cell lines from breast cancer, melanoma and small cell lung cancer have been developed and used successfully. Since the tumor is disintegrated during the procedure, the microanatomy of the tumor microenvironment is lost. Spheroid or organoid generation from these primary tumor cells are of significant interest for the evaluation of patient-specific targets and for screening of drugs in early drug development. The growth of patient-derived cells in 3D cultures as spheroids features physiological relevant cell-cell interactions. In particular, the development of 3D tumor co-cultures from cancer cells in combination with fibroblasts, endothelial cells, immune cells, bone cells or adipocytes enables cross-talk between tumor cells and the stromal cells (140). Tumor organoids have been created from different entities, including colorectal, stomach, liver and pancreas cancers. The use of cryopreserved tumor material, organoids and well-defined patient-derived xenografts from biobank materials is advantageous for drug screening (141).

The complexity and spatial aspects of intra-tumor heterogeneity is reflected best in organotypic tumor tissue slices. In comparison to organoids, organotypic tissue slices retain their natural microenvironment, reflecting the situation of a single patient, and could be regarded as an individual Avatar for this patient tumor response. The tissue is not dissociated and hence tumor cells and tumor microenvironment are maintained in an non-manipulated and autologous condition. Various slicing methods have been described, namely manual choppers, the Krumdieck tissue slicer, and vibratomes. The IMI-funded consortium project PREDECT (http://www.predect.eu) studied slice-explants from a variety of sources. Using slices derived from breast, prostate and lung cancer models, sustained viability of cultured slices was seen for up to 72 h (142). The possibility to compare tissue (tumor) slices from different species is an advantage in translating data from mouse to humans and may help to transfer and validate targets established in mouse models. However, organotypic tissue slices are prepared and cultured heterogeneously using different methods. In principle, a prevalidation study of lung tissue slices showed applicability of suitable standardization (143). Validation for *in vivo* data in co-clinical studies may help to use this tool for efficient P4-medicine (predictive, preventive, personalized and participative). Systemic effects of treatments or metastatic processes in a human setting have been difficult to monitor *in vitro*. However, new developments using human approaches *ex vivo*, to model cancer in microfluidic human organs-on-chip, for instance, promise to identify key molecular, cellular and immunological features of human cancer progression in a fully human setting.

### Patient-Derived Models in Drug Testing and Personalized Medicine

A fast and effective way to evaluate a compound in drug testing or predict responses of a patient to specific anti-cancer drugs is to use high throughput approaches. These procedures are based mainly on simple test models which provide robust data on efficacy and targeted binding of the compound. However, most cell lines lack specific targets and are thus, no longer relevant. Patient-derived cell lines or more complex spheroid containing immune cells help to select candidates at an early stage for preclinical assessment and to provide data for stratified medicine approaches (144). A key step was the development of droplet-based chip platforms encapsulating primary cancer cells in nanoscale spots of hydrogels, allowing for comparison of the *in vitro* data obtained from the chip with clinical data, as well as with gene expression data. In a proof of concept study, the testing of 24 anti-cancer drugs in patient-derived brain cancer cells were well correlated with their oncogene overexpression (c-Met, FGFR1) and *in vivo* xenografts. Further developments use spheroids. Thus, tumor spheroid systems of the PANC-1 cell line in co-culture with pancreatic stellate cells are currently used in minipillar histochips as a tool to analyze stroma-targeting drugs (145).

Extensive preclinical studies are requested by public authorities to demonstrate the efficacy and safety of the test item. The paradigm changes in anti-cancer drug development from “one-size-fits-all” to patient-specific therapies have changed dramatically the requirements for translation to the clinic. Tumor entity driven targets within the cancer cells or in the tumor milieu have made drug testing on simple cancer cell line assays outmoded and demand the development of complex human based immune competent models. Molecular characterization of individual tumors is also paving the way to predictive therapies for individualized medicine. However, these biomarkers, obtained from transcriptomic and proteomic signatures, require evaluation of their predictively in clinical settings. For example, Her2 amplification is a strong predictive marker for trastuzumab treatment of breast cancer, but lacks predictivity in gastric cancer (146). PDX models using patient-derived cells are still the most relevant models to validate biomarkers and tumor relevant targets as they maintain the histopathological features, gene expression profiles, copy number variations and metastatic outgrowth of the original tumors (147). In humanized PDX models, even the testing of drugs targeting immune cells is possible. Safety evaluation for off-target effects, however, needs to be well thought through. New targets regularly arise, which cannot be replicated in animal models nor adequately represented by immune competent models. The implementation of human (tumor) tissue slices may help to gain robust confirmation of the clinical potential of such drugs. Human tumor tissue *ex vivo* reflects the tumor heterogeneity and contains tissue-resident immune cells. It is thus, highly recommended as a validation tool (148). Evidence-based therapy suggestions to clinicians for metastatic cancer is a major challenge since epigenetic changes in cancer cells, altered tumor microenvironment and differences in the cellular composition of the metastatic tissue make it nearly impossible to draw conclusions from therapy predictions made on primary tumors. New technologies enable the detection of circulating tumor cells in easy accessible blood preparations and raise the possibility of characterizing these cells with a more metastatic phenotype and to gain insight into tumor progression (149–151). Inadequate scientific data on early metastatic progression weakens predictivity of therapy options in a metastatic setting. Finally, every model comes with its limitations and test strategies have to be matched to the mode of action of the test item and the individual patient. Integrative test strategies to evaluate efficacy of anti-cancer drugs need the cost-efficient combination of models. Furthermore, the test strategy considers various levels of test complexity as they may be used in a tiered approach.

### Lessons Learned

Test models for immunomodulatory drugs in cancer and autoimmune disease models need to reflect the complexity of the disease. In contrast to immunomodulatory drugs in cancer, treatments for autoimmune diseases are focused on restoring immune tolerance. CAR T cell-derived immunotherapies, chimeric autoantibody receptor T (CAR T) cells, and CAR regulatory T (CAR Treg) cells bring new hope of treatment choices for autoimmune diseases. However, learning from T cell therapy in cancer, attention should be paid to the inflammatory microenvironment in autoimmune disease. Foxp3 expression in the CAR Tregs may be downregulated in this microenvironment and the phenotype may lose its immune suppressive function. It is obvious that there is no single model that reflects all relevant features. However, the use of the Avatar concept could bring significant progress and enable clinical style studies *ex vivo* as well as *in vivo* in humanized mice. While *in-vitro* and *ex-vivo* models usually lack the systemic response and adaptive immune response, murine models are never fully human and always lack a fully human response. Therefore, critical disease mechanisms or therapeutic targets should be validated by a combination of different models which generate reliable and predictive information. First steps have been taken to identify gaps in immune safety assessment within the EU consortium, imSAVAR. Specific modes of actions of immune modulatory drugs are being addressed, for which models or methods to predict adverse immune effects are not available. For this, existing models will be refined and new models and biomarkers developed. A part of the project will be to establish a platform providing biological samples from different resources that can be integrated into the new model systems.

## Conclusion

Our knowledge and understanding of the innate and adaptive immune system currently provides a picture of a multi-component system that is essential for immediate defense against pathogens, as well as for the stimulation of the adaptive immune system. In addition, the constant maintenance of self-tolerance is crucial. However, it is clear that a wide variety of infectious and acquired diseases are closely linked to failure to establish healthy innate immunity. Diseases such as auto-inflammatory diseases are often caused by congenital dysfunction in immune responses. This increased understanding has permitted the development of novel targeted, cell-based therapies and drugs that are now used in normal clinical practice. As our knowledge of the different inhibitory and stimulatory immune mechanisms associated with autoimmune diseases progresses, we shall see significant improvement in early detection and diagnosis, as well as in the use of adequate treatment options.

## Author Contributions

SK, SS, and UK were mainly responsible for the performance of this review. MP acted as a native English specialist in editing the manuscript and also contributed to the content of this manuscript. All authors contributed to the article and approved the submitted version.

## Conflict of Interest

The authors declare that the research was conducted in the absence of any commercial or financial relationships that could be construed as a potential conflict of interest.
